# The Effect of Candesartan Alone and Its Combination With Estrogen on Post-traumatic Brain Injury Outcomes in Female Rats

**DOI:** 10.3389/fnins.2019.01043

**Published:** 2019-12-03

**Authors:** Mojdeh Hajmohammadi, Mohammad Khaksari, Zahra Soltani, Nader Shahrokhi, Hamid Najafipour, Reza Abbasi

**Affiliations:** ^1^Neuroscience Research Center, Neuropharmacology Institute, Kerman University of Medical Sciences, Kerman, Iran; ^2^Endocrinology and Metabolism Research Center, Institute of Basic and Clinical Physiology Sciences, Kerman University of Medical Sciences, Kerman, Iran; ^3^Physiology Research Center, Institute of Neuropharmacology, Kerman University of Medical Sciences, Kerman, Iran; ^4^Cardiovascular Research Center, Institute of Basic and Clinical Physiology Sciences, Kerman University of Medical Sciences, Kerman, Iran

**Keywords:** candesartan, estrogen, intracranial pressure, cerebral perfusion pressure, brain edema, mean arterial pressure

## Abstract

**Aim:** The aim of this study was to evaluate the effect of candesartan (angiotensin II type I receptor blocker) alone and its combination with estrogen on the changes in brain edema, intracranial pressure (ICP), and cerebral perfusion pressure (CPP) following diffuse traumatic brain injury (TBI) in female rats.

**Methods:** TBI was induced in ovariectomized female rats using Marmarou's method. The treatment groups received low-dose (LC) and high-dose (HC) candesartan, estrogen (E2), a combination of estrogen vehicle and candesartan vehicle (oil + vehicle), or a combination of estrogen with low-dose (E2 + LC), or with high-dose (E2 + HC) candesartan. ICP and CPP were measured before and several times after TBI, and the brain water content (brain edema) was measured 24 h after TBI.

**Results:** After the TBI, brain edema and ICP in the estrogen group were lower than in the vehicle and TBI groups. Brain edema and ICP in the HC group were lower than in the vehicle group after TBI. Although there was no significant difference in brain edema and ICP between the LC and vehicle groups, significant differences in these variables were observed when the E2 + LC and E2 + HC groups were compared with the oil + vehicle group after TBI. A significant increase in CPP was observed in the estrogen group 4 and 24 h post-TBI, while this increase was found in the HC and E2 + LC groups 24 h post-TBI.

**Conclusions:** A low dose of candesartan did not exert a protective effect on TBI outcomes, but such an effect did appear after combination with estrogen. This finding suggests that interaction between low-dose candesartan and estrogen improves TBI-induced consequences.

## Introduction

Traumatic brain injury (TBI) is one of the most common causes of death and disability throughout the world (Parchani et al., 2014). Despite extensive efforts, no efficient method has been discovered to treat TBI (Kumar and Loane, 2012). Half of the deaths after TBI are caused by failure to control secondary edema, increase in intracranial pressure (ICP), and reduction in cerebral perfusion pressure (CPP) (Feickert et al., 1999; Stahel et al., 2000), which ultimately lead to brain ischemia. Inflammatory activity (Venero et al., 2004), vascular endothelial cell damage, failure of the blood-brain barrier (BBB) (Meymandi et al., 2018), and up-regulation of adhesion molecules (Khaksari et al., 2017) followed by infiltration of macrophages and T lymphocytes into the brain (Venero et al., 2004) result in brain inflammation (Mofid et al., 2016; Shahrokhi et al., 2016). As a result, efforts to reduce cerebral edema and ICP with the aim of preventing cerebral under-perfusion and ischemia may be an important therapeutic objective to decrease the mortality resulting from TBI. Perhaps one of the reasons for failure in treating TBI is the lack of knowledge of the mechanisms related to the development of brain edema.

Estrogen is currently used to treat brain edema in TBI research. It has been reported that estrogen reduces brain edema and the permeability of the BBB after TBI. Recent studies in our laboratory have shown that estrogen decreases ICP and neurologic outcome after TBI, in which both of its receptors are involved. Other mechanisms have also been proposed for the neuroprotective effects of estrogen after TBI, such as the inhibition of membrane lipid peroxidation (Roof et al., 1994), modulating aquaporin 4 (AQP4) expression (Soltani et al., 2016), matrix metalloproteinase inhibition (MMP9) (Walf et al., 2008), and the production of nitric oxide (NO) (Krause et al., 2002).

Brain structures in BBB contain a large number of angiotensin type 1 receptors (AT1Rs) (Saavedra et al., 2011). Following ischemia, levels of angiotensin 2 (AngII) increase (Ozacmak et al., 2007), which leads to vasoconstriction, increased oxidative stress (Kusaka et al., 2004), vascular endothelial failure and remodeling (Savoia and Schiffrin, 2007), the removal of NO, and apoptosis by AT1R. On the other hand, drugs that inhibit AT1R (angiotensin receptor blockers, ARBs) have neuronal protection, anti-inflammatory, and vasodilatory effects (Benigni et al., 2010). Candesartan is an ARB that crosses the BBB (Groth et al., 2003) and decreases vasoconstriction by blocking AT1R in the cerebral arterioles and possibly via an increase in NO levels. Candesartan blocks oxidative stress by maintaining blood flow (Iwai et al., 2004, 2006) and thereby preventing cerebral edema and the destruction of the BBB. Considering the aforementioned points, drugs that inhibit AT1R receptors can potentially decrease damage following TBI due to their neuroprotective effects against inflammation.

Some studies have reported an interaction between estrogen and Ang II in inflammation processes. Olmesartan (which is an ARB) prevents the reduction of estrogen alpha receptors in the brain of ischemic animals. Furthermore, the neuroprotective effects of ARBs are inhibited by estrogen receptor blockers (Shimada et al., 2011). The anti-inflammatory impact of estrogen on intestinal inflammation after TBI happens by reducing the expression of AngII and AT1R (Chen et al., 2008). Estrogen alpha receptor decreased AngII after cerebral ischemia and thus limited the activation of the renin–angiotensin system (RAS) (Shimada et al., 2011). These results suggest that ARBs and estrogen exert their neuroprotective and anti-inflammatory effects via AngII receptors.

Since few studies have been conducted on the efficiency and anti-inflammatory mechanisms of ARBs alone or in combination with estrogen after TBI, this study was focused on the role of AT1R in diffuse TBI-induced consequences (cerebral edema, ICP, and CPP) by using candesartan. In addition, the role of AT1R in mediating the neuroprotective effects of estrogen was assessed by the administration of estrogen and candesartan alone or their combination in ovariectomized rats with diffuse TBI.

## Materials and Methods

### Experimental Procedure

#### Animals

**Figure 1:**
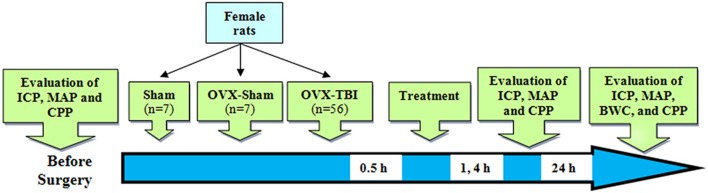
Schematic diagram of the study. Numbers in the arrow show time after TBI. BWC, brain water content; CPP, cerebral perfusion pressure; ICP, intracranial pressure; OVX, ovariectomy; TBI, traumatic brain injury.

Adult female Wistar rats (200–250 g; 12–14 weeks) were kept at a temperature of 20–22 degrees and a light–dark cycle of 12–12 h with free food and water in the animal house of the Afzalipour Faculty of Kerman University of Medical Sciences. The study was carried out under License No. 93/280/K issued by the Ethics Committee of Kerman University of Medical Sciences according to Figure 1. The experiments were performed at the same time of the day in order to exclude the impact of circadian rhythmicity. Anesthesia was performed with ketamine (50 mg/kg) and xylosin (10 mg/kg) (intraperitoneally) before TBI and before each consecutive series of measurements.

#### Drugs

17β-estradiol (E2) and sesame oil were provided by Aburaihan Pharmaceutical Company (Tehran, Iran). Candesartan was purchased from LKT Labs, USA.

#### Experiment Groups

The animals were divided into 10 groups (seven rats in each group), as follows. **Sham–OVX:** female rats whose ovaries were falsely removed and that experienced TBI. **Sham** **+** **OVX:** animals of this group were similar to those of the first group except that their ovaries were removed. **TBI**: rats that experienced TBI 2 weeks after the ovariectomy. **Oil**: ovariectomized rats that intraperitoneally received estrogen vehicle (sesame oil) equal to the amount of the consumed estrogen 30 min after TBI (O'Connor et al., 2005). **E2**: ovariectomized rats that received 1 mg/kg of estrogen intraperitoneally 30 min after TBI (O'Connor et al., 2005). **Veh:** similar to group oil except that they received candesartan vehicle (sodium carbonate 0.1 N) equal to the amount of the consumed candesartan (Panahpour et al., 2013). **Low-dose candesartan (LC)**: ovariectomized rats that received 0.1 mg/kg of candesartan intraperitoneally 30 min after TBI (Tota et al., 2009). **High-dose candesartan (HC)**: similar to group 7 except that they received 0.3 mg/kg of candesartan (Panahpour et al., 2013). **Oil** **+** **Veh:** similar to group 6 except that they received oil + vehicle equal to the amount of candesartan and estrogen received. **E2** **+** **LC:** ovariectomized rats that received candesartan intraperitoneally 30 min after TBI (1 mg/kg, estrogen + 0.1 mg/kg. **E2** **+** **HC:** similar to group 10 except that they received 1 mg/kg of estrogen and 0.3 mg/kg of candesartan.

#### Bilateral Ovariectomy

In order to prevent an estrus cycle and to remove ovarian steroids, the animals were ovariectomized 2 weeks before TBI. After the rats were anesthetized by ketamine (50 mg/kg) and xylosin (10 mg/kg) (intraperitoneally), the lower abdominal area was shaved, and the skin area was cut 2 cm vertically. The tissues and muscles were opened, and fat and intestines were pushed up so that the fallopian tubes could be observed. In each ovary, the fallopian tube was tied in the proximal area by using 0–4 catgut thread, and it was disconnected from the distal area. Finally, after injecting 1–2 ml of isotonic saline, the muscles and skin were sutured, and the stitches were disinfected with iodine. The animals were under care until the end of anesthesia (Wen et al., 2004).

#### Induction of TBI

TBI was diffuse and was created by using the Marmarou method in anesthetized animals after intubation. After putting a metal disc (made of steel with a thickness of 3 mm and a diameter of 10 mm) on the top of the skull (between bregma and lambda) using polyacrylamide glue, the anesthetized animal was put under a TBI device (developed in Kerman Physiology Group). A 250 g weight was released from a distance of 2 m, which landed on the metal disc after passing through a pipe. After TBI, if necessary, the respiration of the animals was immediately brought back by connecting them to a respiratory pump. Finally, the animals were put in a cage and kept under care (Marmarou et al., 1994).

#### Measuring Brain Edema

As an index of brain edema, brain water content was measured 24 h after TBI. After irreversible anesthesia and opening of the skull, the brain was taken out, weighed (wet weight) and dried for 72 h in an incubator at a temperature of 60°C. It was then weighed again (dry weight). The brain water content was calculated using the following formula (Koyama et al., 2004; Khaksari et al., 2018):

(1) $$\begin{array}{l} \text{Brain water content }(\%)=[(\text{wet tissue weight}-\text{dry tissue weight})\\ \;/\text{wet tissue weight}]\times 100 \end{array}$$

#### Measuring ICP

ICP was measured 1 h before and 1, 4, and 24 h after TBI using ICP monitoring system. The anesthetized animal's head was fixed inside a stereotaxic device (Stoeling, USA) (Consiglio and Lucion, 2000), and after locating the cisterna magna area, a needle connected to a saline filed cannula was entered to a depth of 5 mm. The pressure was transferred via the cannula to a transducer, and it was recorded on a Powerlab Physiograph (Software Tutor 4.0, ADInstruments, Australia).

#### Measuring MAP

After the animals were anesthetized, the right carotid artery was cannulated with a polyethylene P50 cannula connected to a pressure transducer and the Power Lab system (ADInstruments, Australia). Systolic and diastolic blood pressures (P_s_ and P_d_, respectively) were recorded regularly, and then MAP was calculated using the following equation. During the pressure recording process, the animals were connected to a respiratory pump (Joukar et al., 2013).

(2) $$\begin{array}{r} \text{MAP}={\text{P}}_{\text{d}}+1/3\;\left({\text{P}}_{\text{s}}-{\text{P}}_{\text{d}}\right) \end{array}$$

#### Determining CPP

CPP was calculated using the following equation (Gilkes and Whitfield, 2007):

(3) $$\begin{array}{r} \text{CPP}=\text{MAP}-\text{ICP} \end{array}$$

#### Statistical Analysis

The results are reported as mean ± SEM. Normal distribution of the data was checked using Shapiro–Wilk's test. If the data were normally distributed, comparison among the groups was performed using one-way analysis of variance (ANOVA) followed by *post-hoc* LSD. If the data were not normally distributed, a non-parametric Kruskal–Wallis test was used. *P* <0.05 was considered as the significance level.

## Results

### Brain Edema

**Figure 2:**
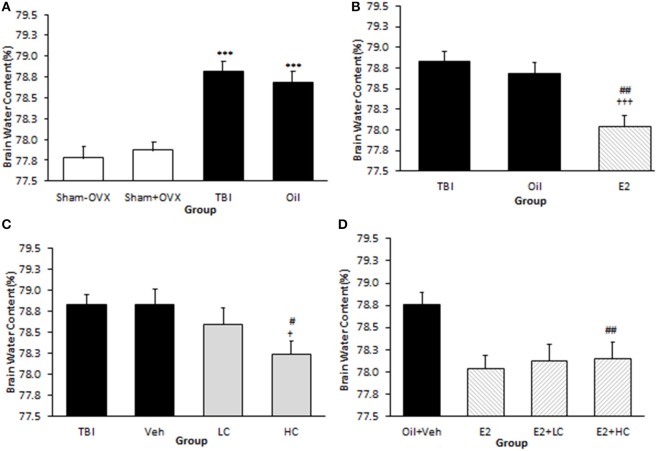
Brain water content (%) in different groups (*n* = 7/group). Data are expressed as mean ± SEM. ***p < 0.001 vs. sham–OVX and sham+OVX **(A)**. ^†††^*p* < 0.001 vs. TBI. ^##^*P* < 0. 01 vs. oil **(B)**. ^†^*p* < 0.05, vs. TBI ^#^*p* < 0.05, vs. Veh **(C)**. ^##^*p* < 0.01 vs. Oil + Veh **(D)**. TBI, traumatic brain injury; oil, estrogen vehicle; E2, estrogen; LC, low-dose candesartan; HC, high-dose candesartan; Veh, candesartan vehicle.

The brain water content in the TBI (78.83 ± 0.12%) and oil (78.69 ± 0.13%) groups was higher than that of the sham (77.87 ± 0.11%) groups (*p* <0.001). However, there was no significant difference between the TBI and oil groups (Figure 2A). The brain water content in the E2 group (78.04 ± 0.14%) was lower than that of the oil group (*p* <0.01) (Figure 2B). The water content in the HC group (78.24 ± 0.15%) was lower than that in the Veh group (78.83 ± 0.18%) (*p* <0.05), while there was no significant difference in brain water content between the LC and HC groups (Figure 2C). Although there was no significant difference in brain edema between the LC and Veh groups, a reduction in brain water content appeared in the E2 + LC and E2 + HC groups compared with the Oil + Veh group (*p* < 0.01). By contrast, edema was not significantly different among E2 + HC, E2 + LC, and E2 groups (Figure 2D).

### ICP Measurement

**Figure 3:**
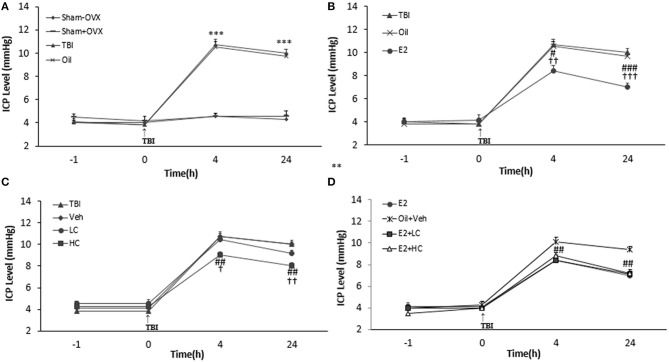
Intracranial pressure (ICP) level in study groups at different times during the study (*n* = 7/group). Results are reported as mean ± SEM. ***p < 0.001 TBI and oil groups compared to sham+OVX and sham–OVX groups 4 and 24 h after TBI **(A)**. ^###^*p* < 0.001 E2 group compared to oil group 24 h after TBI. ^#^*P* < 0.05 E2 group compared to oil group 4 h after TBI. ^†††^*p* < 0.001 E2 group compared to TBI group 24 h after TBI. ^††^*p* < 0.01 E2 group compared to TBI group 4 h after TBI **(B)**. ^##^*p* < 0.01 HC group compared to Veh group 4 and 24 h after TBI. ^††^*p* < 0.01 HC group compared to TBI group 24 h after TBI. ^†^*p* < 0.05 HC group compared to TBI group 4 h after TBI **(C)**. ^##^*p* < 0.01 HC + E2 and LC + E2 groups compared to Oil + Veh group 4 and 24 h after TBI **(D)**. TBI, traumatic brain injury; oil, estrogen vehicle; E2, estrogen; LC, low-dose candesartan; HC, high-dose candesartan; Veh, candesartan vehicle.

There was no significant difference in ICP levels among groups before TBI. However, after TBI, ICP increased in the TBI and oil groups compared to the sham groups at all hours tested (*p* < 0.001) (Figure 3A). The level of ICP significantly decreased in the E2 group in comparison with the oil group at 4 h (8.42 ± 0.48 mm Hg, *p* < 0.05) and 24 h (7 ± 0.37 mm Hg, *p* < 0.001) (Figure 3B) post-TBI. The ICP level decreased in the HC group (9 ± 0.3 and 8 ± 0.3 mm Hg, 4 and 24 h after TBI, respectively) compared to the Veh group (*p* < 0.01) (Figure 3C). There was no significant difference in this index among the E2 and E2 + LC and E2 + HC groups. Although no significant difference in ICP level was observed between the Veh and LC groups at any hour after TBI, the ICP level in the E2 + LC and E2 + HC groups was lower than in the Oil + Veh group 4 and 24 h after TBI (*p* < 0.01) (Figure 3D).

### Measuring Mean Arterial Pressure (MAP)

**Figure 4:**
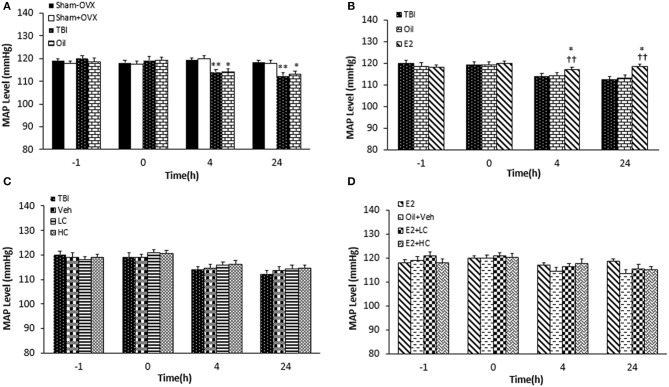
Mean arterial blood pressure (MAP) level in study groups at different times during the study (*n* = 7/group). Results are reported as mean ± SEM. **p < 0.01 TBI group compared to sham+OVX and sham–OVX groups 4 and 24 h after TBI. *p < 0.5 oil group compared to sham+OVX and sham–OVX groups 4 and 24 h after TBI **(A)**. ^††^*p* < 0.01 E2 group compared to TBI group 4 h and 24 h after TBI. *p < 0.5 E2 group compared to oil group 4 and 24 h after TBI **(B)**. No difference observed in MAP among TBI, Veh, LC, and HC groups at 4 and 24h after TBI **(C)**. No significant difference in MAP among groups at any time during the study **(D)**. TBI, traumatic brain injury; oil, estrogen vehicle; E2, estrogen; LC, low-dose candesartan; HC, high-dose candesartan; Veh, candesartan vehicle.

Before the TBI, there was no significant difference among the groups regarding MAP. MAP significantly decreased in the TBI and oil groups compared to the sham+OVX and sham–OVX groups 4 and 24 h after the trauma (Figure 4A). While no significant difference in MAP was reported between the oil and E2 groups 4 h after TBI, MAP increased in the E2 group (118.57 ± 0.94 mm Hg) compared to the TBI and oil groups (*p* < 0.05 and *p* < 0.01, respectively) 4 and 24 h after TBI (Figure 4B). In rats treated with candesartan (Figure 4C), no difference in MAP was observed among the TBI, Veh, LC, and HC groups at 4 and 24 h after the TBI. In Figure 4D, there was no significant difference in MAP among the groups at any time during the study.

### Measuring CPP

**Figure 5:**
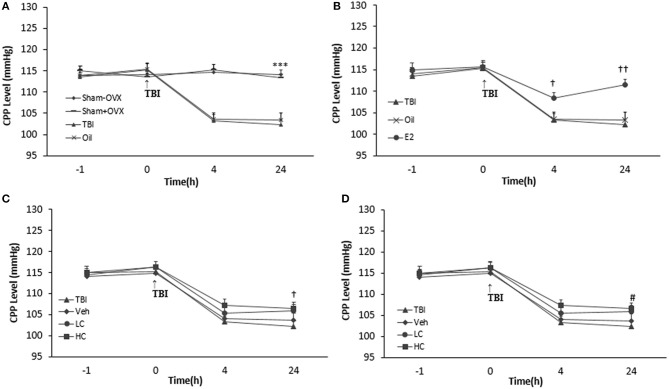
Cerebral perfusion pressure (CPP) level in study groups at different times during the study (*n* = 7/group). The results are reported as mean ± SEM. ***p < 0.001 TBI and oil groups compared to sham+OVX and sham–OVX groups 4 and 24 h after TBI **(A)**. ^#^*p* < 0.05 E2 group compared to oil 4 h after TBI. ^†^*p* < 0.05 E2 group compared to oil group 4 h after TBI. ^††^*p* < 0.01 E2 group compared to oil group 24 h after TBI **(B)**. ^†^*p* < 0.05 HC group compared to TBI group 24 h after TBI **(C)**. ^#^*p* < 0.05 E2 + LC group compared to Oil + Veh group 24 h after TBI **(D)**. TBI, traumatic brain injury; oil, estrogen vehicle; E2, estrogen; LC, low-dose candesartan; HC, high-dose candesartan; Veh, candesartan vehicle.

There was no significant difference among the groups before TBI. CPP decreased significantly in the TBI (103.28 ± 1.42 and 102.28 ± 1.22 mm Hg 4 and 24 h after TBI, respectively) and oil (103.57 ± 1.49 and 103.42 ± 1.64 mm Hg 4 and 24 h after TBI, respectively) groups compared to the sham+OVX group (115.28 ± 1.14 and 113.42 ± 0.94 mm Hg 4 and 24 h after TBI, respectively) (*p* < 0.001) (Figure 5A). There was an increase in the CPP level of the E2 group relative to the oil group (Figure 5B), 4 h (108.42 ± 1.28 mm Hg, *P* < 0.05) and 24 h (111.57 ± 1.23 mm Hg, *P* < 0.001) after TBI.

Although the differences in CPP between groups treated with candesartan (Figure 5C) were not significant in comparison with between other groups 4 h after TBI, the increase in the HC group (106.57 ± 1.32 mm Hg) was significant compared to the TBI group 24 h after TBI (*P* < 0.05). Meanwhile, no significant difference was observed among the HC, LC (105.85 ± 1.54 mm Hg), and Veh groups (103.71 ± 1.67 mm Hg). No significant difference in CPP appeared between the E2 + HC and Oil + Veh groups 4 and 24 h after TBI. Although there was no significant difference in CPP between the E2 + LC and E2 + Veh groups 4 h after TBI, the E2 + LC group presented a significant increase compared to the Oil + Veh group (104.14 ± 1.59 mm Hg) 24 h later (*p* < 0.05). The CPP index values of the E2, E2 + LC, and E2 + HC groups did not differ at any time after TBI (Figure 5D).

## Discussion

In previous studies by our group, it was shown that estrogen has a neuroprotective effect following TBI. Studies have indicated that RAS and AT1R increase damage after cerebral ischemia (Shimada et al., 2011). In this study, we considered the hypothesis that taking an AT1R blocker (candesartan) in combination with estrogen could play a role in estrogen-induced neuroprotection after TBI. The main findings of our study were: (1) estrogen decreases brain edema and ICP and increases CPP after TBI. (2) A high dose of candesartan decreases brain edema and ICP and increases CPP after TBI. (3) The combination of candesartan and estrogen had no reinforcing effect on the above changes to indices induced by estrogen.

In the present study, TBI caused an increase of 1.23% in brain water content (brain edema) in the traumatic group in comparison with the sham+OVX group. Estrogen decreased this index by ~0.83% compared with the oil group, which indicates an estrogen anti-edema neuroprotective effect after TBI. The anti-edema mechanism of estrogen may be through reducing oxidative stress (Behl et al., 1997) and pro-inflammatory cytokines or increasing anti-inflammatory cytokines, reducing AQP4 (Soltani et al., 2016) and apoptosis activity (Brown et al., 2009).

Our findings are in agreement with our recent experiments. These findings are also in line with other studies that showed that the administration of E2 significantly decreased brain water content (O'Connor et al., 2005) in TBI and ischemic cerebral injury (Shin et al., 2011).

Another part of the study showed that TBI increased ICP and that this lasted for 24 h after the trauma. On the other hand, estrogen reduced ICP 4 and 24 h after TBI. Estrogen prevented the increase in ICP 4 and 24 h after TBI compared to the Veh group. The effect of estrogen on ICP is in line with previous studies reporting that estrogen decreased ICP 4 and 24 h after TBI. Although this study showed that estrogen had a neuroprotective effect after TBI, this neuroprotective effect of estrogen has not been reported in some other studies (Carswell et al., 2004; Bingham et al., 2005). The differences in these findings may be due to differences in the severity and model of injury, drug dose, and species of animals.

The reasons for the reduction of ICP by estrogen after TBI include: reduction of brain edema and AQP4 expression (Soltani et al., 2016), increase in cerebral blood flow (Hurn et al., 1995), and prevention of BBB destruction after TBI. Furthermore, one could also refer to the increase in antioxidant activity (Sugioka et al., 1987), adjustment in the production of NO (Krause et al., 2002), stimulation of estrogen alpha receptors in catecholaminergic neurons of the nucleus tractus solitarius (Shughrue et al., 1997) followed by hypotension (Chan and Sawchenko, 1994), increased secretion of arginine vasopressin and oxytocin (Mecawi et al., 2011), and reduction of inflammatory cytokines (Soltani et al., 2016). In this study, a direct correlation was found between brain edema and the ICP level 24 h after injury (*p* < 0.001, R = 0.5). Therefore, it could be inferred that the changes in ICP are partly due to the changes in brain water content.

One of the therapies that can be used in TBI patients is the maintenance of sufficient CPP (Rosner et al., 1995). In another part of the study, CPP was calculated, indicating a significant decrease in the TBI group 4 and 24 h after TBI compared to that in the sham+OVX group. Furthermore, compared to the Veh group, estrogen could prevent the drop in CPP 4 and 24 h after TBI by about 4.68 and 7.88%, respectively. MAP and ICP are two factors that affect CPP, and MAP measurement in the estrogen group showed that CPP is only greater than in the Veh group in the 24th hour. Therefore, it can be concluded that the increase in CPP by estrogen in the fourth hour compared to the Veh group is caused only by the reduction of ICP by estrogen. However, the increase in CPP after 24 h was due to MAP increase and ICP reduction. It seems that the effect of estrogen on ICP reduction was greater than its effect on MAP increase. Estrogen in healthy animals (non-traumatic) with normal blood pressure did not increase blood pressure since our study showed that blood pressure in ovariectomized animals was no different from in sham animals.

The findings of this study are consistent with other studies that showed a reduction in the CPP after TBI and demonstrated its increase by administration of estrogen. Furthermore, other studies have shown that the low CPP causes more damage to the brain (Rooker et al., 2003).

Our study showed that blood pressure decreases 4 and 24 h after trauma, which is in line with another study that reported that blood pressure decreases after TBI and that this decrease increases damage to neurons (Engelborghs et al., 1998). Rooker et al. proposed that the decrease in blood pressure after TBI could lead to the increase in ICP and cerebral edema (Rooker et al., 2003). The increase in blood pressure by estrogen is in agreement with another study (Araujo et al., 2013).

TBI leads to hypoxia in the brain through cerebral hypoperfusion (Rosner et al., 1995) and decrease in CBF (Engelborghs et al., 2000). If an agent can decrease brain hypoperfusion via increasing cerebral blood flow or blood pressure, it is a neuroprotective agent. Since it has been reported that cerebral ischemia caused by Ang II is mediated through AT1R (Michel et al., 2013) and that, on the other hand, AT1R is responsible for the central and peripheral activities of Ang II (Benicky et al., 2011), the effect of candesartan, an AT1R receptor blocker, was investigated in another part of this study. It was found that a single dose of 0.3 mg/kg candesartan reduced cerebral edema by about 75% after the TBI. Furthermore, this compound decreased ICP 4 and 24 h after TBI by ~15.97 and 20%, respectively. On the other hand, candesartan increased CPP 24 h after the TBI by about 2.75%.

The results of this study are in line with another study suggesting a reduction of neuroinflammation in astrocytes and microglia by candesartan (Lanz et al., 2010). The findings also confirm other studies that pointed to an increase in cerebral blood flow by an AT1R blocker (Engelhorn et al., 2004) and to the protective and anti-ischemic effect of 0.3 mg/kg of candesartan (Faure et al., 2008).

The mechanisms through which candesartan exerts its neuroprotective effect (reduction of brain edema and ICP) include: increase in micro-vascular circulation, increased expression of endothelial NOS (eNOS) in cerebral arteries (Liu et al., 2008), reduction of inflammation (Zhou et al., 2005), decrease in oxidative stress, reduction of TGFβ1 (Villapol et al., 2012), control and release of norepinephrine (Nap et al., 2003), inhibition of inflammatory signals that reach the brain through the vagus (Quan and Banks, 2007), and improvement of baroreflex sensitivity (Kishi et al., 2012). Another possible neuroprotective mechanism of candesartan could be through AT2R (Liu et al., 2008) or peroxisome proliferator-activated receptor-gamma receptor (PPAR-γ agonist) (An et al., 2010).

A further useful effect of candesartan is CPP increase. Given that two factors are effective in increasing CPP, the current study showed that blood pressure was not affected by the dose of consumed candesartan. There were also inverse correlations of brain edema and ICP level with CPP level 24 h after trauma (*p* < 0.01, R = −0.5; *p* < 0.001, R = −0.72, respectively) and also between ICP level and CPP level 4 h after injury (*p* < 0.001, R = −0.64) in the current study. Thus, the increase in CPP was due to the decrement in ICP. Contrasting results have been reported by other researchers, such as a decrease in blood pressure in response to AT1R blockers in the day after administration (Castillo et al., 2004).

The results of this study showed that although taking estrogen combined with different doses of candesartan did not change the response induced by a high dose of candesartan, it did produce a response with a low dose of candesartan. Reduction of brain edema in E2 + LC was not significantly different from that in E2, and this means that estrogen modulates Ang II activity. Estrogen probably strengthens the candesartan signaling pathway through AT1R. This also means that when we have to consume candesartan, estrogen can also be used against cerebral edema.

The findings of this study are in line with a study by Almeida-Pereira that showed that estrogen caused insensitivity of the AT1R receptor (Almeida-Pereira et al., 2016). Our results are also confirmed by a different study that suggests that estrogen strengthens the neuroprotection created by an AT1R blocker via alpha receptor (Shimada et al., 2011).

Possible mechanisms by which candesartan reinforces the effects of estrogen include: weakening the AT1R response to estrogen (Ciriello and Roder, 2013), increasing the expression of AT2R receptors (Yoshimura et al., 1996), up-regulation of angiotensin-converting enzyme 2 (ACE2) (Shimada et al., 2011), and increasing the MASS receptor expression reduction leading to raising Ang (1-7) and strengthening of the signaling pathway Ang (1-7) (Cheng et al., 2015).

The results showed that findings pertinent to ICP are similar to the results for brain edema in combination groups. In other words, a low dose of candesartan had no effect on ICP, but it reduced ICP in the E2 + LC group. The mechanism by which candesartan strengthens the effect of estrogen includes the same mechanisms as mentioned for brain edema.

The CPP results for combination groups showed that LC was not effective in increasing CPP, yet, after combining it with estrogen, such an increase was obtained and the results became similar to brain edema.

It seems that the therapeutic effect of estrogen and candesartan is the reduction of brain edema, and this decreases ICP and increases CPP (O'Connor et al., 2005). Reducing cerebral edema decreases regional pressure on tissues and increases CPP after TBI. Increased ICP leads to brain inflammation, and thus the reduction of ICP by these compounds decreases brain edema.

## Conclusion

The results of this study showed that the neuroprotective effects of candesartan (an AT1R blocker) were dose-dependent, that only a high dose of candesartan had impacts similar to estrogen, and that this effect was applied directly without the intermediation of estrogen. The combination of estrogen and a low (non-effective) dose of candesartan induced a neuroprotective effect, implying that the interaction between estrogen activity and AngII probably exists at the AT1R level.

It is suggested that future research be carried out on a molecular and biochemical level to precisely determine the estrogen-strengthening mechanism that induces a neuroprotective effect with a low dose of candesartan after TBI.

## Ethics Statement

The animal research was carried out under License No. 93/280/K issued by the Ethics Committee of Kerman University of Medical Sciences.

## Author Contributions

MK and HN directed the project, contributed to the interpretations, and prepared the manuscript. MH and RA carried out study evaluations. ZS and NS supervised and directed the project and carried out the interpretations. All of the authors read and approved the final manuscript.

### Conflict of Interest

The authors declare that the research was conducted in the absence of any commercial or financial relationships that could be construed as a potential conflict of interest.

## Abbreviations

AngII: angiotensin 2
AQP4: aquaporin 4
ARB: angiotensin receptor blocker
AT1R: angiotensin receptor type 1
AT2R: angiotensin receptor type 2
BBB: blood–brain barrier
CPP: cerebral perfusion pressure
E2: 17β-estradiol
ICP: intracranial pressure
MAP: mean arterial pressure
NO: nitric oxide
RAS: renin–angiotensin system
TBI: traumatic brain injury.
